# Serum Visinin-Like Protein 1 Is a Better Biomarker Than Neuron-Specific Enolase for Seizure-Induced Neuronal Injury: A Prospective and Observational Study

**DOI:** 10.3389/fneur.2020.567587

**Published:** 2020-09-25

**Authors:** Zheren Tan, Jianlin Jiang, Fafa Tian, Jinxin Peng, Zhiquan Yang, Shuyu Li, Xiaoyan Long

**Affiliations:** ^1^Department of Neurology, Xiangya Hospital, Central South University, Changsha, China; ^2^Department of Neurosurgery, Xiangya Hospital, Central South University, Changsha, China; ^3^National Clinical Research Center for Geriatric Disorders, Xiangya Hospital, Central South University, Changsha, China

**Keywords:** biomarker, visinin-like protein 1, caveolin-1, neuron specific enolase, neuronal injury, epilepsy

## Abstract

**Introduction:** Visinin-like protein 1 (VILIP-1) is an established biomarker of neuronal injury. The levels of serum VILIP-1, neuron-specific enolase (NSE) and caveolin-1 (CAV-1) were measured to investigate potential of VILIP-1 as a biomarker for seizure-induced neuronal injury, and the correlation of VILIP-1 with severity of epilepsy and blood-brain barrier dysfunction were investigated.

**Materials and Methods:** Patient with epilepsy from 14 to 70 years of age and age-, sex-matched healthy subjects were involved in this study. All blood sample of patients were collected within 3–72 h after the seizure. The severity of epilepsy and levels of serum VILIP-1, NSE and CAV-1 were measured. Accuracy of VILIP-1 and NSE was obtained from receiver operating curve analyses. Associations between VILIP-1 and severity of epilepsy, VILIP-1 and CAV-1 were investigated.

**Results:** A total of 58 patients and 29 healthy control subjects were included in our study. The levels of serum VILIP-1, NSE, and CAV-1 in the patient group were significantly higher than those in the control group. VILIP-1 has higher and significant accuracy for assessing seizure-induced neuronal injury compared with NSE. VILIP-1 levels were positively associated with severity of epilepsy and CAV-1 in patients with epilepsy.

**Conclusions:** VILIP-1 may be a better serum biomarker than NSE for assessing seizure-induced neuronal injury and even brain injury caused by various pathological condition. Further studies are required to explore the clinical contribution of VILIP-1 in diagnosis, treatment strategies and outcome assessments of epilepsy.

## Introduction

Epilepsy is one of the most common chronic neurological disorders. It affects ~0.7% of the population (1). Previous studies have confirmed recurrent epileptic seizures may lead to neuronal injury in patients, that could induce subsequent irreversible neurologic structural changes, further development of epilepsy (2, 3). A reliable biomarker that reflects seizure-induced neuronal injury may be used to study mechanisms of epilepsy, predict seizure outcome, identify patients in need of more appropriate and aggressive neuroprotective strategies and evaluate its efficacy.

Various biomarkers have been studied on seizure-induced neuronal injury, such as Neuron-specific enolase (NSE), S100 calcium binding protein B(S100B), Ubiquitin Carboxy-Terminal Hydrolase L1(UCH-L1), microtubule-associated protein 2(MAP-2), tau protein, and so on (3–8). Among all, NSE is the most widely investigated and applied biochemical markers in assessing the neuronal injury, and can be measured in serum and CSF. Previous studies in humans have found increased NSE in cerebrospinal fluid (CSF) and serum associated with epileptic seizures (2, 3, 9). Besides, NSE levels were associated with seizure severity (3, 10).

However, NSE was also found in red cells and platelets (11, 12), and the hemolysis can lead to NSE increase in blood, which may affect the accuracy and clinical value of serum NSE (sNSE) as a biomarker (13). Moreover, lumbar puncture is invasive, not systematically performed in routine clinical care after seizure attack, and cannot be easily repeated for hours- or days-long kinetic monitoring and follow-up. So that it is significant to find a better blood biomarker for seizure-induced neuronal injury with high sensitivity and specificity.

Visinin-like protein 1(VILIP-1) is a neuron-specific calcium sensor protein strongly expressed in the central nervous system(CNS) (14), and penetrates into CSF after destruction of brain cells (15, 16), which is originally studied as a stroke marker and identified as a useful marker of neuronal injury in stroke, Alzheimer's disease and traumatic brain injuries (16–19). However, the utility of VILIP-1 as a useful biomarker of seizure-induced neuronal injury and comparison of efficacy between VILIP-1 and NSE have not been investigated in epilepsy.

Previous studies had confirmed the Blood-brain barrier (BBB) dysfunction caused by epilepsy (20, 21), which may provide a promotion of extravasation of brain-specific proteins into peripheral blood (22). Caveolin-1 (CAV1) is an integral membrane protein, mainly expressed in vessels in the CNS (23) and play an important role in protection of BBB (24). In the study conducted by Liu et al. the CAV-1 increased with the BBB hyperpermeability in three mainstream BBB dysfunctional model *in vivo* (25). Another study has clearly demonstrated that the CAV-1 expression paralleled the breakdown of the BBB in rats following the ischemic injury in ischemic rat model (26). Those findings imply that peripheral assessment of BBB dysfunction induced by seizure could be achieved by detection of serum CAV-1(sCAV-1).

The primary aim of the study was to investigate the potential of serum VILIP-1(sVILIP-1) as a biomarker for seizure-induced neuronal injury. Therefore, we compared sVILIP-1 levels with sNSE levels in patients with epilepsy and controls. In addition, CAV-1 were tested to provide information on the correlation between sVILIP-1 levels and BBB dysfunction.

## Materials and Methods

### Study Population

This prospective observational study was conducted in the Xiangya Hospital, Central South University, Changsha, China, from February 2017 to November 2017. The inclusion criteria were the following: (1) patients with a confirmed diagnosis of epilepsy according to the latest criteria of the International League Against Epilepsy for classification of seizures (27). (2) no history of stroke, traumatic brain injuries, CNS infections, metabolic disease and brain space occupying lesion in the last 3 months (3) no history of diseases that could affect the level of sVILIP-1, sNSE, and sCAV-1 (e.g., Alzheimer's disease, motor neuron disease, traumatic brain injuries, multiple sclerosis, etc.). (4) no combination with severe diseases affected internal organs (e.g., heart, liver, kidney and lung, etc.) and severe infection. (5) no combination with systemic disease (e.g., tumor, cachexia, etc.). (6) the availability of complete clinical data and laboratory findings. (7) age is ranged from 14 to 70 years.

Severity of epilepsy was graded by the Chalfont-National Hospital Seizure Severity Scale (NHS3). Twenty-nine age-, sex-matched healthy subjects (15 men, 14 women; mean age, 34.52 ± 8.82 years) served as the control group. None of the subjects in the control group had any systemic or neurologic illness, history of head trauma, or any family history of neurologic diseases. The protocol for this study was approved by the Research Ethics Committee of the Xiangya Hospital. Written informed consent was obtained from all subjects before their enrollment.

All samples were obtained within 3–72 h after the seizure. Patient group was divided into four patient subgroups according to the interval between sample collection and end of seizure: A (3–12 h); B (12–24 h); C (24–48 h); D (48–72 h).

### Biochemical Procedures

Five ml of blood was collected and centrifuged at 3,500 rpm for 10 min. Haemolysed samples were excluded because of possible false elevations of sNSE caused by hemolysis. After separating serum, the serum samples were stored at −80°C till analysis.

Levels of sVILIP-1 were determined using a double-antibody sandwich human VILIP-1 enzyme-linked Immuno Sorbent Assay (ELISA) kit (BioVendor, Brno, Czech Republic). Levels of sNSE were determined using Electrical chemiluminescent immunoassay. Immunoassay analysis was performed by analyzer (Roche Cobas6000 E601). Levels of sCAV-1 were determined using a double-antibody sandwich human CAV-1 ELISA kit (CUSABIO, Wuhan, China). All samples were analyzed at the same time and all procedures were performed according to the manufacturer's instructions.

### Statistical Analysis

Data processing and analyses were conducted using SPSS 22.0 for Windows (IBM, Armonk, New York). Mean and standard deviations were calculated for parametric variables and medians and quartiles for non-parametric variables. According to data distribution, two-sample *t*-test (parametric) and Mann Whitney *U*-test (non-parametric) were used to account for two groups comparison; Kruskal-Wallis test followed by Bonferroni Procedure (non-parametric) was used to account for multiple comparisons; correlation between sVILIP-1 and sCAV-1 levels were assessed using Spearman rank correlation coefficient and scatter plot. To determine clinical ability of sVILIP-1 to assess seizure-induced neuronal injury, receiver-operating characteristic (ROC) curve analyses were conducted and the area under the curve (AUC) with 95% CI that evaluates the sensitivity and specificity of biomarker were calculated. All tests were two-sided and statistical significance was determined at *p* < 0.05.

## Results

Overall, a total of 58 patients with epilepsy meeting the selection criteria and 29 healthy control subjects were included in the present study. Patient group and control group were comparable at baseline without any significant difference in age and gender. Demographics and clinical features of all groups were shown in Table 1. There was no significant difference among patients with respect to gender, history of CNS diseases and administration of AED on the level of serum biomarker (*p* > 0.05 for all).

**Table 1:** Demographic and clinical profile of study population.

**Variable**	**Patient group**	**Control group**
Age (yr, *x* ± *s*)	32.25 ± 12.37	34.52 ± 8.82
Gender, M/F	32/26	15/14
Interval between sample collection and end of seizure (*n*)		—
3–12 h	10	
12–24 h	22	
24–48 h	17	
48–72 h	9	
History of CNS diseases (*n*, %)	19, 33%	—
Stroke	2	
Febrile seizures	3	
Meningoencephalitis	3	
Traumatic brain injuries	11	
NHS3 score (*n*, %)		—
≤ 10	27, 47%	
> 10	31, 53%	
Age at seizure onset (yr, *x* ± *s*)	21.13 ± 13.85	—
Duration of epilepsy (yr, *x* ± *s*)	11.11 ± 8.99	—
Administration of AED (n)		—
Monotherapy	27	
Carbamazepine	10	
Valproic acid	6	
Oxcarbazepine	8	
Topiramate	1	
Phenytoin	1	
Levetiracetam	1	
Combination therapy	14	
Others	9	
None	8	

*CNS, Central nervous system; AED, Anti-epileptic drugs; —, indicates there is no value*.

The levels of sVILIP-1, sNSE, and sCAV-1 in patient group and control group are shown in Table 2. The levels of sVILIP-1, sNSE and sCAV-1 in patient group were significantly higher than those in the control group (*p* < 0.001 for all, Table 2). In order to investigate the change of serum biomarker levels in different time periods after end of seizure, multiple comparisons of the levels of all biomarkers were performed between the four patient subgroups (A, B, C, D groups) and control group. It was clearly demonstrated that The levels of sVILIP-1 in all patient subgroups were significantly higher than those in the control group (A vs. Control, *p* = 0.003; B vs. Control, *p* < 0.001; C vs. Control, *p* < 0.001; D vs. Control, *p* = 0.001, Table 3). The levels of sNSE in B group and C group were significantly higher than those in the control group (B vs. Control, *p* = 0.01; C vs. Control, *p* = 0.019; Table 3). The levels of sCAV-1 in all patient subgroups were significantly higher than those in the control group (A vs. Control, *p* = 0.007; B vs. Control, *p* = 0.001; C vs. Control, *p* < 0.001; D vs. Control, *p* < 0.001, Table 3). No significant differences were observed among the four patient subgroups with respect to the levels of all biomarkers (*p* > 0.05 for all).

**Table 2:** The levels of sVILIP-1, sNSE and sCAV-1 between patient group and control group.

	**Patient group** **(*n* = 58)**	**Control group** **(*n* = 29)**	***P*-value**
sVILIP-1 (pg/ml)	73.94 (53.66, 97.74)	38.77 (32.59, 44.90)	*P* < 0.001
sCav-1 (pg/ml)	127.81 (98.16, 189.45)	54.26 (28.76, 71.89)	*P* < 0.001
sNSE (ng/ml)	5.34 (3.88, 7.13)	3.52 (2.77, 5.04)	*P* < 0.001

*Data represented as median (interquartile range)*.

**Table 3:** The levels of sVILIP-1, sNSE, and sCAV-1 among four patient subgroups and control group.

**Groups**	**Cases**	**sVILIP-1 (pg/ml)**	**sNSE (ng/ml)**	**sCAV-1 (pg/ml)**
Control group	29	38.77 (32.59, 44.90)	3.52 (2.77, 5.04)	54.26 (28.76, 71.89)
A group	10	61.67 (53.15, 82.18)^**#**^	4.67 (3.64, 5.98)	108.84 (90.96, 186.12)^**#**^
B group	22	77.90 (56.17, 92.34)^**#**^	5.47 (3.94, 6.90)^*^	132.14 (110.32, 187.43)^**#**^
C group	17	70.97 (51.98, 107.35)^**#**^	5.16 (3.89, 8.57)^*^	130.84 (84.98, 232.47)^**#**^
D group	9	79.55 (56.67, 100.84)^**#**^	4.48 (3.75, 7.66)	130.84 (95.59, 231.82)^**#**^

Patient group was divided into 4 subgroups according to the interval between sample collection and end of seizure: A (3–12 h), B (12–24 h), C (24–48 h), D (48–72 h); Data represented as median (interquartile range);

# *P < 0.01*,

* *P < 0.05, compared with control group*.

**Figure 1:**
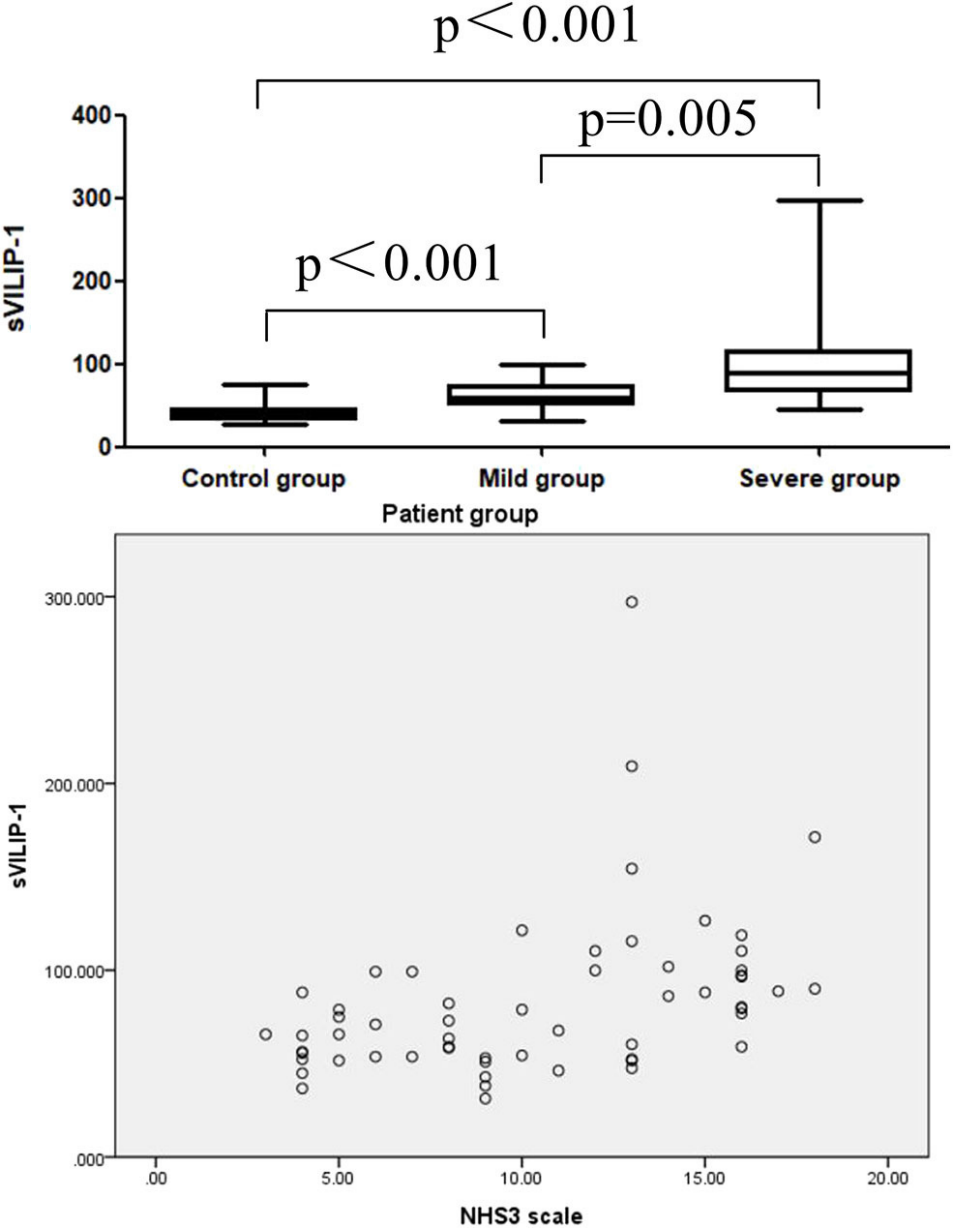
Boxplot for pair-wise comparison of sVILIP-1 levels and related scatter plot between sVILIP-1 levels and NHS3 scale in patient group. sVILIP-1, serum visinin-like protein 1; NHS3 scale, chalfont-National Hospital Seizure Severity Scale.

To investigated the correlation between severity of epilepsy and the levels of sVILIP-1, all patients was divided into severe group (NHS3 > 10, *n* = 31) and mild group(NHS3 ≤ 10, *n* = 27), the multiple comparisons were performed between severe group and mild group and control group. It was shown that levels of sVILIP-1 in severe group were higher than that in mild group and control group (severe vs. mild, *p* = 0.005; severe vs. control, *p* < 0.001, Figure 1), and the levels of sVILIP-1 in mild group were higher than that in control group (*p* < 0.001, Figure 1). In addition, there was a positive correlation between sVILIP-1 and HNS3 scale in patients with epilepsy (*p* < 0.001, Figure 1).

**Figure 2:**
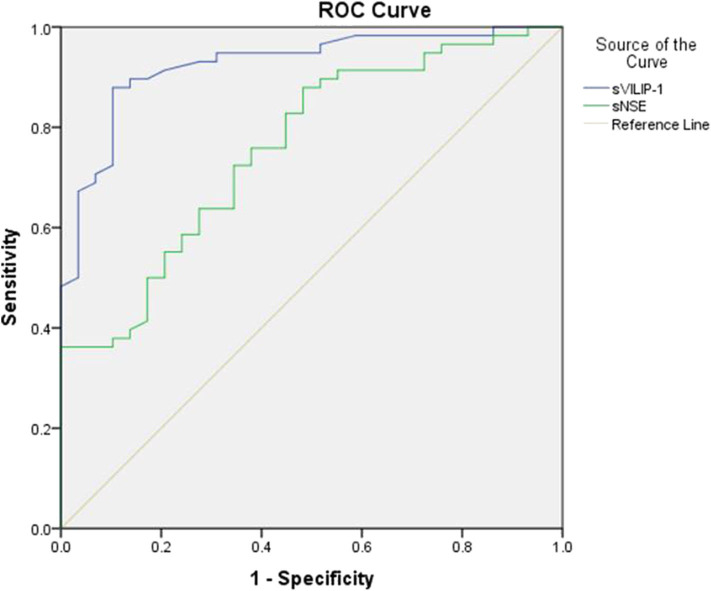
ROC analyses of sVILIP-1 an sNSE. ROC analyses, receiver-operating characteristic curve analyses; sVILIP-1, serum visinin-like protein 1; sNSE, neuron-specific enolase.

ROC analyses were performed to test the utility of sVILIP-1 and sNSE. Serum VILIP-1 and sNSE had significant accuracy for assessing seizure-induced neuronal injury (Table 4 and Figure 1). Binary logistic regression did not show that interaction between sVILIP-1 and sNSE nor sNSE had significant predictive value on the likelihood of seizure-induced neuronal injury (*p* > 0.05 for both). Compared with sNSE (specificity of 51.72%, sensitivity of 89.7%), sVILIP-1 (specificity of 89.7%, sensitivity of 87.9%) provided higher accuracy (*p* < 0.002, Figure 2).

**Table 4:** AUC of serum biomarker for assessing seizure-induced neuronal injury.

	**sVILIP-1**	**sNSE**
AUC (95% CI)	0.93 (0.85–0.97)	0.76 (0.66–0.84)
*P*-value	*P* < 0.001	*P* < 0.001

*AUC, Area under the curve; CI, Confidence interval*.

**Figure 3:**
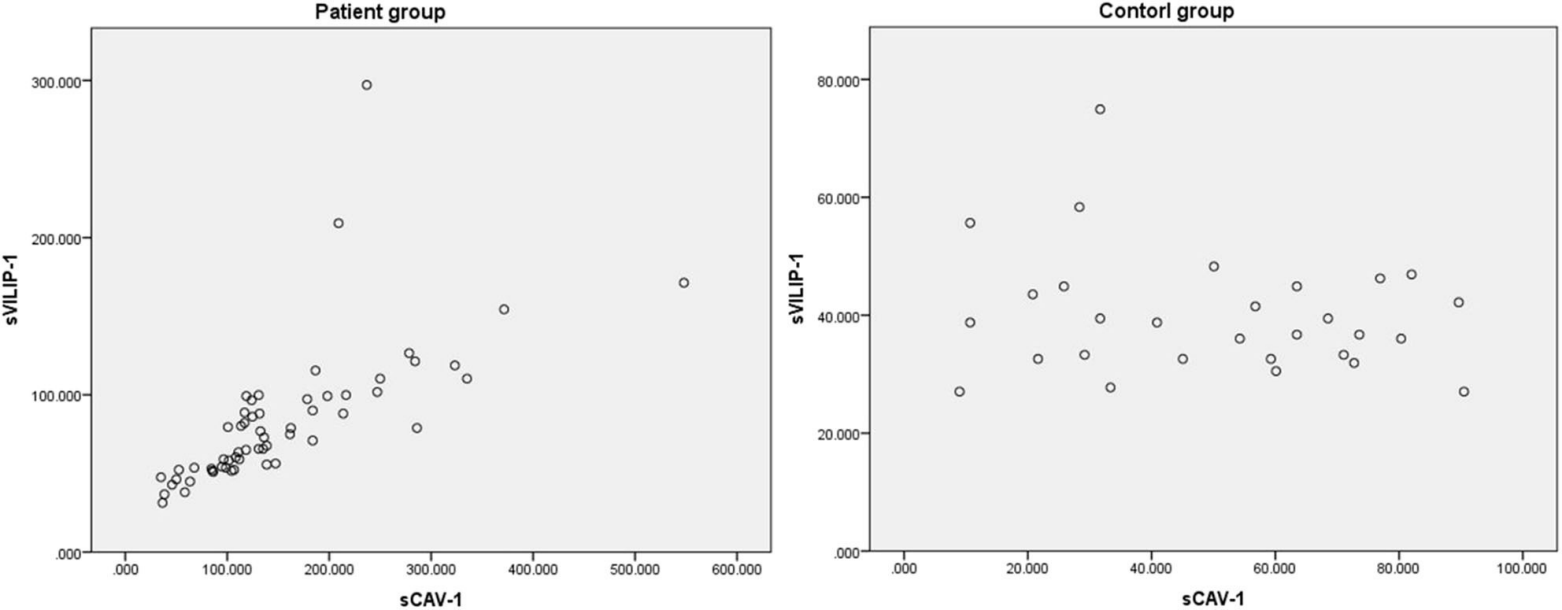
Related scatter plot between levels of sVILIP-1 and sCAV-1. sVILIP-1, serum visinin-like protein 1; sCAV-1, serum caveolin-1.

In addition, there was a positive correlation between levels of sVILIP-1 and sCAV-1 in patient group (*n* = 58, *r* = 0.869, *P* < 0.001, Figure 3); no correlation between levels of sVILIP-1 and sCAV-1 was observed in control group (*n* = 29, *r* = −0.105, *P* = 0.587, Figure 3).

## Discussion

NSE is a highly specific marker for neurons and peripheral neuroendocrine cells, so that increase in NSE has been used as a reliable biomarker in diagnosis, prognosis and follow-up of various diseases, such as cancer (lung cancer, neuroendocrine tumors), Guillain-Barré syndrome and Creutzfeldt-Jakob disease, etc. (28, 29). In addition, Increased sNSE have also been found associated with brain damage, and it has been widely applied in basic researches (30, 31) and the process of diagnosis and assessment of prognosis in stroke, epilepsy, traumatic brain injuries, Encephalopathy, etc. (28, 32–36). In the present study, sNSE levels increased within 72 h after seizures, which is consistent with those of previous studies that found a correlation between increased NSE and epileptic seizures (2, 3, 9).

VILIP-1, a member of NCS protein family, is abundantly found in neurons and be involved in various pathological disturbances of Ca2+ homeostasis leading to neuronal loss (18), and it was released into CSF and blood after destruction of neurons (15, 16). The utility of VILIP-1 as a biomarker of neuronal injury was confirmed in numerous studies. Compared with control subjects, increased serum or CSF levels of VILIP-1 has been observed in Alzheimer's disease, stroke, and traumatic brain injuries (16–19, 37).

In the present study, the levels of sVILIP-1 and sNSE were significantly increased within 72 h after epileptic seizures, those findings suggested that the seizure induced neuronal injury occurs in the pathological process of epileptic seizures. Moreover, levels of sVILIP-1 in severe group were higher than that in the mild group and control group, sVILIP-1 levels were associated with NHS3 scale, those results indicated that sVILIP-1 levels were associated with seizure severity.

To the authors' knowledge, this is the first study to evaluate the utility of serum VILIP-1 for seizure-induced neuronal injury and made a comparison of efficacy between VILIP-1 and NSE. In the present study, the sVILIP-1 levels increased significantly 3–72 h after seizure, but sNSE levels increased significantly 12–48 h after seizure, which means the change of sVILIP-1 can be discovered earlier and has a longer period to be tested for clinical work compared with sNSE. Besides that, ROC analyses demonstrated that sVILIP-1(specificity of 89.7%, sensitivity of 87.9%) provided higher accuracy compared with sNSE (specificity of 51.72%, sensitivity of 89.7%), all those findings suggest that sVILIP-1 is a promising and reliable biomarker for assessing seizure-induced neuronal injury and are more accurate and feasible that sNSE. Some patients with psychological disorders, whose clinical presentation resemble an epileptic seizure, will be misdiagnosed as epilepsy because of inaccurate or incomplete descriptions of symptoms and normal findings of neuroimaging and interictal electroencephalography. So that, sVILIP-1 could be useful for differential diagnosis between real seizures and psychogenic non-epileptic seizures. Moreover, the increase in sVILIP-1 level indicated the presence and severity of seizure-induced neuronal injury. Therefore, clinicians could reduce neuronal injury as early as possible with administration of neuroprotective agents. Further studies are required to investigate the sVILIP-1 levels within 3 h after seizure, which may optimize the timing of neuroprotective therapy.

Several previous studies confirm that CAV-1 could be used as a marker for assessing the BBB dysfunction (25, 26). In the present study, sVILIP-1 levels in physiological conditions are negligible, therefore, the release into the peripheral blood must occur due to BBB dysfunction, which was confirmed by the elevation of sCAV-1. In addition, we found a significant positive correlation between sVILIP-1 levels and the sCAV-1 level in the patient group, but not in the control group. It means that patients with a higher severity of seizure-induced neuronal injury have more severe BBB dysfunction.

The main limitations of our study should be noted. These include the single-center design, relatively small sample size, and heterogeneity among the patient groups with respect to interval between the penultimate seizure and last seizure, multiple seizures in short period before sample collection may impact the levels of biomarkers. Further studies are required to examine the CSF VILIP-1 level in epilepsy, combination of sVILIP-1 levels with that of CSF levels would have helped us to assess seizure-induced neuronal injury more accurately.

## Conclusions

In conclusion, the present study found sVILIP-1 levels increased after epileptic seizure compared with control subjects, accuracy and utility of sVILIP-1 are higher than sNSE, which indicated that sVILIP-1 may be a better biomarker for assessing seizure-induced neuronal injury and even brain injury caused by various pathological condition. Further studies are required to explore the clinical contribution of VILIP-1 in diagnosis, treatment strategies and outcome assessments of epilepsy.

## Data Availability Statement

The raw data supporting the conclusions of this article will be made available by the authors, without undue reservation.

## Ethics Statement

The studies involving human participants were reviewed and approved by Research Ethics Committee of the Xiangya Hospital. Written informed consent to participate in this study was provided by the participants' legal guardian/next of kin.

## Author Contributions

ZT, JJ, and FT: conceptualization. ZT and JJ: methodology. ZT, JJ, JP, and ZY: investigation. ZT: formal analysis, resources, writing, and original draft. SL and XL: writing-review & editing and Supervision. All authors have read and approved the manuscript. All authors had full access to all the data in the study, took responsibility for the integrity of the data, and the accuracy of the data analysis.

## Conflict of Interest

The authors declare that the research was conducted in the absence of any commercial or financial relationships that could be construed as a potential conflict of interest.
